# Improvement of cardiac function by mesenchymal stem cells derived extracellular vesicles through targeting miR-497/Smad7 axis

**DOI:** 10.18632/aging.203533

**Published:** 2021-09-16

**Authors:** Min Chen, Jianfei Chen, Caiting Li, Ranjie Yu, Weiwen Chen, Cunrong Chen

**Affiliations:** 1 Department of Critical Care Medicine, Union Hospital of Fujian Medical University, Fuzhou 350001, Fujian, China; 2 Department of Critical Care Medicine, Affiliated Hospital of Putian University, Putian 351100, Fujian, China; 3 Department of Intensive Care Unit, Quan Zhou First Hospital Affiliated to Fujian Medical University, Quanzhou 362000, Fujian, China

**Keywords:** cardiac function, mesenchymal stem cells, extracellular vesicles, miR-497, Smad7

## Abstract

Background: The extracellular vesicles (EVs) secreted by bone marrow mesenchymal stromal cells (MSCs) have the ability to improve Myocardial infarction (MI). Some microRNAs (miRNAs) including miR-497 and related target genes have been proved to be closely linked with heart diseases. However, EVs could regulate MI process through miR-497, and the mechanisms have not been fully reported.

Methods: Ligation of left anterior descending artery was performed to established MI animals model. Hypoxia cell model was established through lowering the level of oxygen. The cell invasion, migration, and proliferation were measured using tanswell, wound heating, and MTT assays. HE, Masson trichrome, and Sirius Red staining were used to investigate the morphological changes.

Results: Overexpression of miR-497 reversed the promotion of cell migration, invasion, and proliferation caused by EVs. The improvement of cardiac function induced by EVs could also be reversed by overexpression of miR-497. Direct binding site between Smad7 and miR-497 was identified. Knockdown of Smad7 reversed the improvement of cardiac function induced by EVs.

Conclusions: We found that EVs isolated from MSCs might improve the cardiac injury caused by MI through targeting miR497/Smad7. This study provides novel potential therapeutic thought for the prevention and treatment of MI through targeting miR-497/Smad7.

## INTRODUCTION

Myocardial infarction (MI) is characterized by high mortality and morbidity globally [1, 2]. In the past decades, the improvement of prevention and treatment strategies have greatly decreased the death caused by MI [3, 4]. However, MI is still a major health problem worldwide. More specific prevention and treatment methods need to be explored to solve this issue.

It was reported that bone marrow mesenchymal stromal cells (MSCs) could accelerate the cardiac function recovery of MI animals by decreasing fibrosis and infarct area [5, 6]. Meanwhile, MSCs could inhibit the damaging effects of the cytokine storm induced by COVID-19 on the heart and cardiovascular system [7]. MSCs have been widely used in many fields [8, 9]. The extracellular vesicles (EVs) secreted by MSCs have been believed to play an important role during the repair process. EVs could carry microRNAs (miRNAs), chemokines, cytokines, and growth factors, might exert specific effects through these subjects [10].

miRNAs consist of 20-25 nucleotides, and they could accelerate mRNA degradation or suppress mRNA translation. Through these methods, miRNAs could negatively affect the expression of specific genes [11]. miRNAs are proved to be closely linked with many kinds of pathophysiological processes including MI, tumor, diabetes, and atherosclerosis [12]. Previous studies indicated that miR-497 was a tumor inhibitor in many types of cancers including cervical cancer, gastric cancer, and lung cancer [13, 14]. In addition, the regulation roles of miR-497 in heart diseases were also reported. For example, miR-497 might regulate the anoxia/reoxygenation injury through affecting autophagy and apoptosis, and miR-497 was proved to be a potential biomarker of MI [15, 16]. However, if EVs secreted from MSCs could improve cardiac function through targeting miR-497 has not been reported.

Smad family is considered to be a major regulator of TGF-β signaling pathway. Smad family includes inhibitory Smad, receptor-mediated Smad, and common regulator Smad [17]. Smad7 belongs to inhibitory Smad, and it was reported to be highly expressed in prostate cancer, melanoma, and thyroid carcinoma. Smad7 interacts physically with TGF-β receptor and suppresses the activation of TGF-β [18]. Smad7 was reported to be involved in the regulation of MI by miR-21 [19]. Meanwhile, Smad7 was proved to be a downstream target of miR-497 regulating breast cancer [20]. However, if Smad7 is involved in the repair of cardiac function induced by EVs and miR-497 have not been investigated.

In this study, hypoxia cell model and MI animal model were established. Overexpression of miR-497 and knockdown of Smad7 vectors were constructed. The improvement of cardiac function by EVs through miR-497/Smad7 axis was validated. The present study might provide a new thought for the prevention and treatment of MI injury.

## RESULTS

### Identification of EVs and measurement of miR-497 in the hypoxia cells

**Figure 1:**
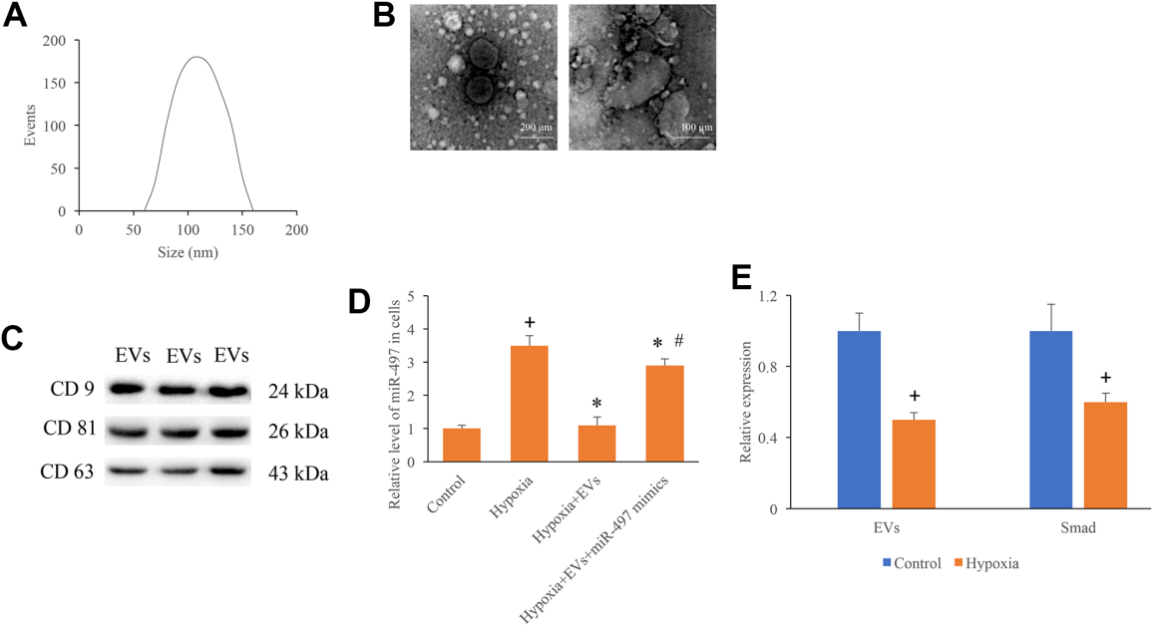
**Identification of EVs and measurement of miR-497 in the hypoxia cells.** (**A**) The size distribution of EVs; (**B**) Morphology of EVs measured by an electron microscope; (**C**) Specific markers of EVs were measured using western blotting; (**D**) The mRNA level of miR-497 was measured after different treatments; (**E**) The levels of EVs and Smad7 were significantly suppressed after hypoxia treatment. + indicated P<0.05 compared with the group control. * indicated P<0.05 compared with the group hypoxia. # indicated P<0.05 compared with group hypoxia+EVs.

The size distribution, scanning electron microscopy images, and surface markers of isolated EVs were investigated. The average diameter peak at 110 nm in DLS was observed (Figure 1A). The homogeneous and spheroidal vesicles of EVs could observed through scanning electron microscopy images (Figure 1B). In addition, the specific markers of EVs, CD9, CD81, and CD63, were identified (Figure 1C). Meanwhile, after establishment of hypoxia cell model, the expression of miR-497 was measured. The mRNA level of miR-497 was remarkably increased after hypoxia treatment, but miR-497 was significantly suppressed after EVs treatment (Figure 1D). In addition, the levels of EVs and Smad7 were significantly suppressed after hypoxia treatment (Figure 1E).

### Overexpression of miR-497 reversed the promotion of cell migration, invasion, and proliferation caused by EVs

**Figure 2:**
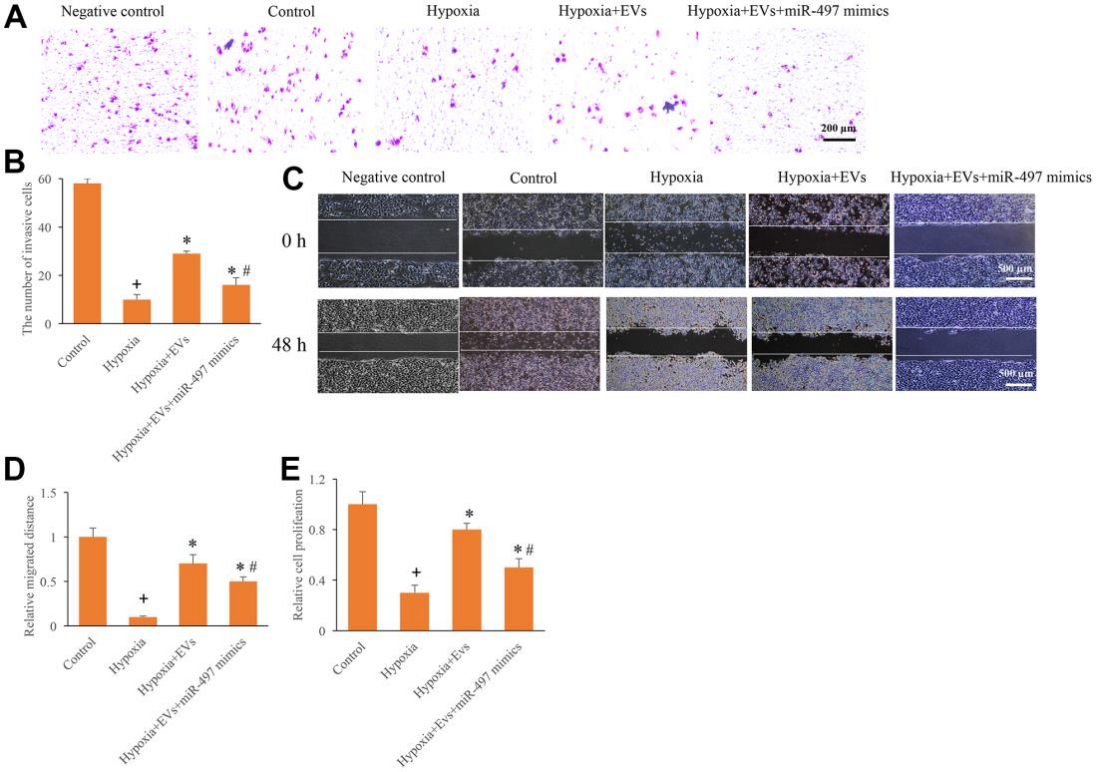
**Overexpression of miR-497 reversed the promotion of cell migration, invasion, and proliferation caused by EVs.** (**A**) Influence of EVs and miR-497 mimics on cell invasion; (**B**) The influence of EVs and miR-497 mimics on cell invasion was analyzed; (**C**) Influence of EVs and miR-497 mimics on cell migration; (**D**) The influence of EVs and miR-497 mimics on cell migration was analyzed; (**E**) I The influence of EVs and miR-497 mimics on cell proliferation was analyzed. + indicated P<0.05 compared with the group control. * indicated P<0.05 compared with the group hypoxia. # indicated P<0.05 compared with group hypoxia+EVs.

To investigate the role of EVs and miR-497 on the cell migration, invasion, and proliferation, the hypoxia cell model was established. After hypoxia administration, cell migration, invasion, and proliferation were markedly inhibited (Figure 2A–2E). However, the EVs treatment remarkably increased the cell migration, invasion, and proliferation ability after hypoxia treatment. In addition, simultaneous with miR-497 mimics and EVs significantly reversed the influence of EVs, and overexpression of miR-497 significantly suppressed the cell migration, invasion, and proliferation ability (Figure 2A–2E). Therefore, EVs and miR-497 might be involved in the regulation cell viability in the hypoxia cell model.

### Overexpression of miR-497 reversed the improvement of cardiac function induced by EVs

**Figure 3:**
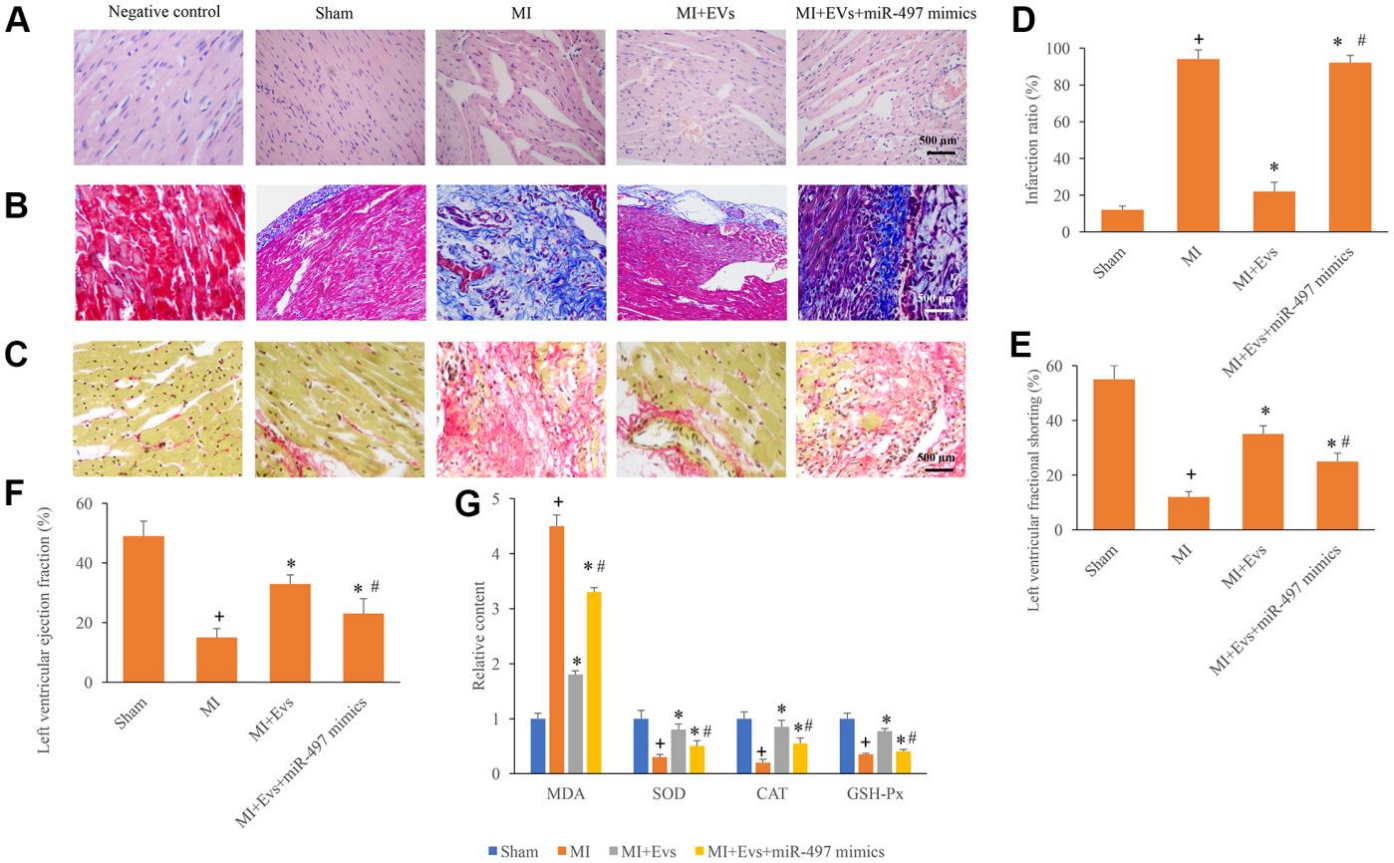
**Overexpression of miR-497 reversed the improvement of cardiac function induced by EVs.** (**A**) Influence of EVs and miR-497 mimics on the histological changes of MI animals; (**B**) Collagen deposition was investigated using Masson trichrome staining; (**C**) Collagen deposition was investigated using sirius red staining; (**D**) Influence of EVs and miR-497 mimics on infarction was analyzed; (**E**) Influence of EVs and miR-497 mimics on left ventricular fractional shortening was analyzed; (**F**) Influence of EVs and miR-497 mimics on left ventricular ejection fraction was analyzed; (**G**) Influence of EVs and miR-497 mimics on MDA, SOD, CAT, and GSH-Px was analyzed. + indicated P<0.05 compared with the group sham. * indicated P<0.05 compared with the group MI. # indicated P<0.05 compared with group MI+EVs.

HE, Masson, and Sirius Red Staining were used in this study to investigate the influence of EVs and miR-497 on the morphological changes of MI rats. Well-arranged and possessed intact muscle fibers were observed in the group sham and MI+EVs through HE staining (Figure 3A). However, remarkable necrosis and irregularly arranged muscle fibers were found in the group MI and MI+EVs+miR-497 mimics (Figure 3A). Meanwhile, the results of Sirius red and Masson staining indicated that markedly increase of collagen deposition and infarction ratio could be observed in the group MI and MI+EVs+miR-497 mimics (Figure 3B–3D). However, obvious decrease of collagen deposition and infarction ratio were found in the group EVs (Figure 3B–3D). EVs also significantly promoted the levels of left ventricular ejection fraction and left ventricular fractional shortening (Figure 3E, 3F) compared with group MI. The levels of left ventricular ejection fraction and left ventricular fractional shortening were suppressed markedly by overexpression of miR-497 (Figure 3E, 3F). The levels of MDA, SOD, CAT, and GSH-Px in the serum were also investigated. In the group MI+EVs, the content of MDA was significantly suppressed, but the levels of SOD, CAT, and GSH-Px were remarkably increased compared with group MI (Figure 3G). However, simultaneous treatment with miR-497 mimics markedly reversed the influence of EVs indicating that EVs might improve cardiac function through targeting miR-497.

### Direct binding site between Smad7 and miR-497 was identified

**Figure 4:**
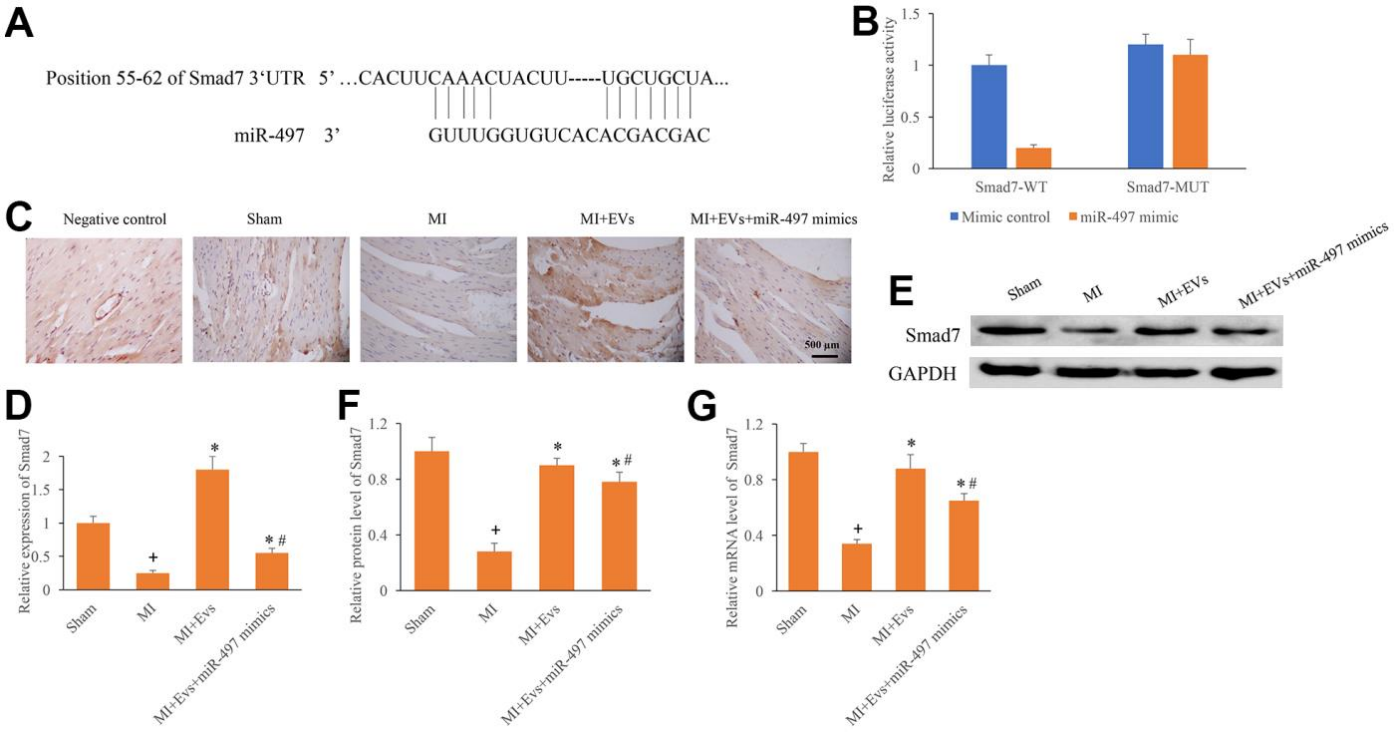
**Direct binding site between Smad7 and miR-497 was identified.** (**A**) The binding site between Smad7 and miR-497 was predicted; (**B**) The binding site between Smad7 and miR-497 was verified; (**C**) Influence of EVs and miR-497 mimics on the expression of Smad7 was measured using IHC staining; (**D**) Influence of EVs and miR-497 mimics on the expression of Smad7 was analyzed; (**E**) Influence of EVs and miR-497 mimics on the expression of Smad7 was measured using western blotting; (**F**) Influence of EVs and miR-497 mimics on the protein expression of Smad7 was analyzed; (**G**) Influence of EVs and miR-497 mimics on the mRNA expression of Smad7 was analyzed. + indicated P<0.05 compared with the group sham. * indicated P<0.05 compared with the group MI. # indicated P<0.05 compared with group MI+EVs.

The potential binding target of miR-497 was predicted and identified. The direct binding site between miR-497 and Smad7 was identified (Figure 4A, 4B). In addition, the expression of Smad7 was measured using IHC, western blotting, and PCR methods. We found that the expression of Smad7 in the group MI was remarkably inhibited compared with group sham. However, EVs significantly promoted the expression of Smad7, but the increased of Smad7 could be reversed by overexpression of miR-497 (Figure 4C–4G). Therefore, EVs might regulate the repair of cardiac function in MI rats through targeting miR-497/Smad7 axis.

### Knockdown of Smad7 reversed the improvement of cardiac function induced by EVs

**Figure 5:**
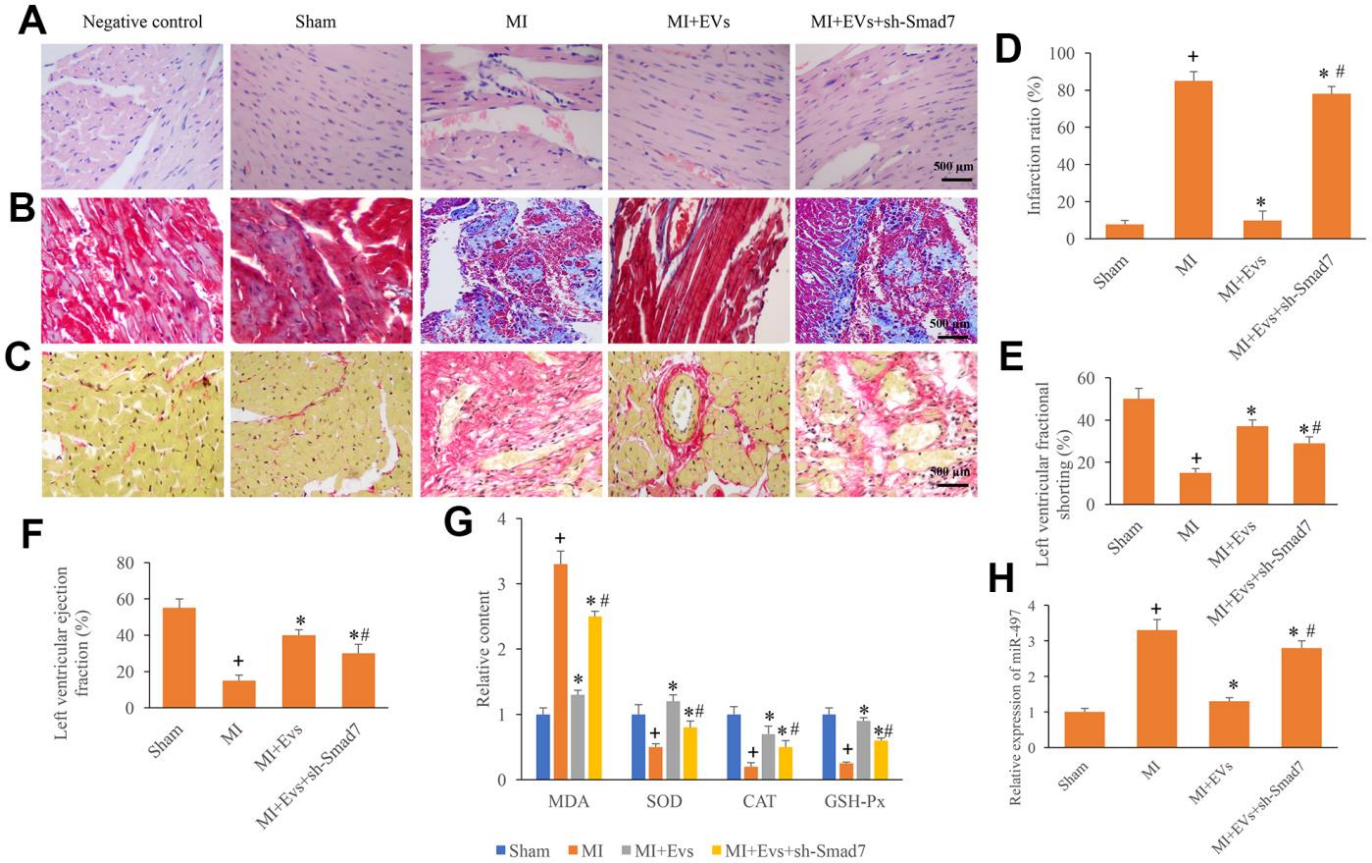
**Knockdown of Smad7 reversed the improvement of cardiac function induced by EVs.** (**A**) Influence of EVs and sh-Smad7 on the histological changes of MI animals; (**B**) Collagen deposition was investigated using Masson trichrome staining; (**C**) Collagen deposition was investigated using sirius red staining; (**D**) Influence of EVs and sh-Smad7 on infarction was analyzed; (**E**) Influence of EVs and sh-Smad7 on left ventricular fractional shortening was analyzed; (**F**) Influence of EVs and sh-Smad7 on left ventricular ejection fraction was analyzed; (**G**) Influence of EVs and sh-Smad7 on MDA, SOD, CAT, and GSH-Px was analyzed; (**H**) sh-Smad7 reversed the influence of EVs on the expression of miR497. + indicated P<0.05 compared with the group sham. * indicated P<0.05 compared with the group MI. # indicated P<0.05 compared with group MI+EVs.

To further unfold the role of Smad7 in the process of MI, knockdown of Smad7 was constructed. We found that EVs could significantly improve the myocardial remodeling, decrease the level of collagen deposition and infarction ratio (Figure 5A–5D). However, knockdown of Smad7 remarkably reversed the influence of EVs, and increased the levels of collagen deposition and infarction ratio (Figure 5A–5D). Meanwhile, the increase of left ventricular ejection fraction and left ventricular fractional shortening induced by EVs were also suppressed by sh-Smad7 (Figure 5E, 5F). Similar influencing trends were observed in terms of the contents of MDA, SOD, CAT, and GSH-Px in the serum. In the group MI+EVs, the expression of MDA was significantly inhibited, but the levels of SOD, CAT, and GSH-Px were remarkably increased compared with group MI (Figure 5G). However, simultaneous treatment with sh-Smad7 markedly reversed the influence of EVs. Meanwhile, we found that sh-Smad7 could reverse the influence of EVs on the expression of miR497 (Figure 5H). Therefore, Smad7 might be one potential function target of EVs on miR497. These findings indicate that EVs might improve cardiac function through targeting miR-497/Smad7 axis.

## DISCUSSION

Despite of great improvement of treatments strategies, MI is still one of the leading death cause in the world. MI is characterized by increase of cardiac fibroblasts, extracellular matrix, and cardiomyocytes loss. MI is a major complication of coronary artery disease. Severe hypoxia in the myocardium tissues and thrombotic occlusion of coronary arteries are the main causes of MI. MI could further result in structure change of artery vascular and cardiac function damage. Exploring novel therapeutic strategies is urgent for the treatment of MI.

Stem cell therapy can reduce the infarct area of MI, and stem cell-derived EVs may be the main carrier for stem cells to play the role of MI protection [21]. The secreted EVs contains lipids, proteins, messenger RNA, microRNA, and they are important carriers for intercellular transmission of information through anti-apoptosis, anti-fibrosis, and anti-inflammatory. EVs could also promote endothelial cell proliferation and angiogenesis during MI [22]. In this study, we found that EVs could significantly decrease the MI damage, and this protection effect might be achieved through miR-497/Smad7 axis.

Many studies have confirmed that miRNAs are closely linked with several heart diseases including MI. For example, miR-21 could mediate the cardiac fibrosis of MI rats [19]. miRNAs could act as molecular sponges to regulate the target genes. Meanwhile, some miRNAs such as miR-15b, miR-20b-5p, and miR-21 have been proved to target Smad7, and thereby affecting MI process.

Previous studies indicated that the level of cardiac troponin I, a biomarker of MI, was significantly positively correlated with the content of miR-497 in the serum [23]. Meanwhile, it was reported that exercise training could improve the cardiac function through suppressing the expression of miR-497 in the heart tissues after MI [24]. Our data suggested that overexpression of miR-497 could aggravate the cardiac function damage after MI, which is in line with previous study.

Our study indicated that miR-497 could directly target the 3’UTR of Smad7, and further influence the expression of Smad7. EVs could increase the expression of Smad7, but overexpression of miR-497 markedly inhibited the levels of Smad7 (Figure 4C–4G). Furthermore, downregulation of Smad7 could reverse the influence of EVs on cardiac function, and accelerate the cardiac damage after MI (Figure 5A–5F). These findings indicated that EVs might exert cardiac protective function through targeting Smad7. Smad7 was believed to be an attenuator of TGF-β. Smad7 could suppress the phosphorylation caused by TGF-β, which is a key regulator of fibrosis progression. Therefore, the influence of EVs and miR-497 on the TGF-β needs to be further explored.

The elevated level of ROS after MI is another damage factor for cardiac function. High level of ROS could lead to oxidative stress, which further induce cell apoptosis of cardiomyocyte. The regulation of redox enzymes by EVs through miR-497/Smad7 should be one potential cardio-protective mechanism. In addition, it was reported that VEGF could promote the proliferation of vascular endothelial cells and induce angiogenesis, and VEGF has a protective effect on MI rats [25]. Collagen type 1 and α- SMA are important markers of myocardial fibrosis, and the high expression of them could promote cellular fibrosis [26]. It is reported that collagen type 1, α- SMA, and TGF- β were remarkably increased in the MI model. However, down regulation of them can reduce the dysfunction of mitochondria and lysosomes in MI rats [27]. Therefore, if EVs/miR-497/Smad7 could affect MI through regulating VEGF, collagen type 1, and α- SMA might be the potential function mechanism.

In summary, we found that EVs isolated from MSCs might improve the cardiac injury caused by MI through targeting miR497/Smad7. This study provides novel potential therapeutic thought for the prevention and treatment of MI through targeting miR-497/Smad7.

## MATERIALS AND METHODS

### Isolation and identification of EVs

The umbilical cord blood derived MSCs were cultivated in MEM medium (Gibco, USA) containing 5 FBS (Gibco, USA) and 1 mM GlutaMAX (Gibco, USA). The EVs were isolated and identified as described previously [28]. Three tandem runs of centrifugation (2300 g for 20 min, 2300 g for 20 min, and 100000 g for 1 h, Optima L-100XP, USA) were conducted to centrifuge cells. Size distribution and morphology were measured using dynamic light scattering and scanning electron microscopy, respectively. The surface markers (CD9, CD81, and CD63) were measured using western blotting.

### Cell culture and hypoxia treatment

H9c2 cardiomyocytes (#CRL-1446) purchased from ATCC were used in this study. The H9c2 cardiomyocytes were cultured using DMEM containing 5% FBS, 50 μg/ml streptomycin, 0.05mM bromodeoxyuridine, and 50 U/ml penicillin in an atmosphere composed of 5% CO_2_ at 37° C. Hypoxia treatment was performed via culturing cells in an atmosphere composed of 94% N_2_, 2% O_2_, and 4% CO_2_ for 24 h. Then, the cells were used for different experiments.

### Construction of miR-497 overexpression and Smad7 knockdown vectors

The miR-497 mimics, sh-Smad7, and relative negative controls were purchased from Ribobio (Guangzhou, China). Virus solution (6×10^12^ genome-containing particles) was used in the animal experiment through caudal vein injection. Lipofectamine 2000 reagent was used for transfection according to the instruction.

### Animal experiment

Wistar rats purchased from laboratory animal center of university and technology of China were used in this study. Male rats (220 g-240 g) were raised in the cage with free access to water and food. The cage environment was kept on 24 - 26° C and 40 - 50% humidity. Left anterior descending ligation was conducted to establish MI model. Before ligation, the animals were anesthetized using xylazine (12 mg/kg) and ketamine (120 mg/kg) through intraperitoneal injection. The animal chests were firstly shaved, and the thoracic cavities were opened. Ligation of left anterior descending was maintained for 1 h. The rats in the sham group were conducted with thoracic cavity open operation but without left anterior descending artery ligation. 10 days later, the animals were treated with EVs, EVs+miR-497 mimics, EVs+sh-Smad7. After 10 days, the animals were sacrificed and heart tissues were isolated for histological examination. All experiments were approved by Ethic Committee of Affiliated Hospital of Putian University (approval reference number: PT202066).

### Real-time polymerase chain reaction (RT-PCR)

Total RNA from heart tissues and cells was isolated with Trizol reagent (Aidlab Biotechnology, China). 50 ng RNA was reverse-transcribed using SuperScript RT kit (Qiagen, USA) for RNA. SYBR Premix Ex TaqTM II kit (Takara, China) was used to perform RT-PCR. GAPDH (forward: 5′-CCTTCCGTGTTCCTACCCC-3′ and reverse: 5′-GCCCAGGATGCCCTTTAGTG-3′) was used as an internal control. The relative mRNA expression of Smad7 (forward: 5′-TGTCCAGACGCTGTACCTTCCT-3′ and reverse: 5′-AGTCTTCTCCTCCCAGTATGCCA-3′) and miR-497 (forward: 5′- GTGCAGGGTCCGAGGT-3′ and reverse: 5′-GCTTCGGCAGCACATATACTAAAAT-3′) were calculated.

### Western blotting

Tissues and cells were lysed firstly, and protein content was measured using BCA method (Nanjing Jiancheng, China). 15 μg protein samples were separated on 8% SDS-PAGE and transferred onto nitrocellulose membrane. After blocking with 5% fat free milk for 30 min, the membranes were cultivated with primary antibodies (1:1000) at 4° C overnight. After washing with PBS, the membranes were cultured with secondary antibody (1:1500). Then, immune-reactive proteins were measured. The protein bands were analyzed through ImageJ software.

### Transwell measurement

Firstly, the lower chamber was supplied with 500 μL DMEM containing 5% FBS. Cells (1×10^5^) suspended with 150 μL DMEM without FBS were plated into the upper chamber. After 24 h, 4% polyformaldehyde was applied to fix cells. Then, the cells in the lower chamber were stained using Giemsa (Sigma, USA). Three random fields were selected for analysis.

### Wound healing measurement

H9c2 cardiomyocytes were suspended with DMEM and seeded into a 12-well plate. When cell confluence reached 80%, a wound was made using a 1 mL pipette. After 48 h, the relative migrated distance was calculated.

### Ventricular function measurement

The hemodynamic assessment of left ventricular function was measured with Xario ultrasound device (Toshiba, Japan). After anaesthesia, the hemodynamic items of rats were detected using MP100-CE (BIOPAC Systems, USA).

### HE, Masson trichrome, and sirius red staining

After animal sacrifice, the heart tissues were isolated and fixed using 4% paraformaldehyde (Beyotime, Beijing, China) for 24 h. Then, the tissues were embedded using paraffin and cut into 4-μm thick sections. After de-paraffin, the heart tissues were stained using HE, Masson trichrome, and sirius red staining methods, respectively. The stained sections were captured using an inverted optical microscope (Olympus, Japan).

### Immunohistochemistry staining

The tissue slides were treated with de-paraffin, antigen repair (microwave heating, 10 s), washing (PBS, 5 min), 5% H_2_O_2_ (3 min), blocking (5% goat serum, 10 min), and washing (PBS, 5 min). Then, the sections were incubated with anti-Smad7 antibody overnight at 4° C. After washing with PBS twice for 3 min, the sections were incubated with secondary antibody for 2 h at room temperature. Finally, the slides were captured using an inverted optical microscope (Olympus, Japan).

### Cell proliferation

Cells were seeded into 96-well plate at a density of 1×10^5^/well, and cultivated for 24 h. After different treatments, cells were washed twice with PBS. Then, cells were cultured with 20 μL MTT solution (#ST316, Beyotime, China) for 4 h. After adding 100 μl dimethyl sulfoxide, OD at 490 nm was measured.

### Luciferase reporter assay

The umbilical cord blood derived MSCs were inoculated firstly into 6-well plates, and transfection reagents were prepared as described below: Smad7-WT plasmid/Smad7-MUT plasmid were transfected with miR-NC/miR-342-3p mimic plasmid using Lipofectamine 2000 (Invitrogen, USA). After 48 h, the dual-luciferase reported gene (Renilla and Firefly) was detected. Firefly luciferase activity was normalized against Renilla luciferase activity.

### Statistical analysis

SPSS 20.0 was used for statistical analysis in this study. Standard t-test was used to compare data between two groups. p<0.05 was considered to be statistical significance.
